# Effect of Immune Reconstitution on the Incidence of HIV-Related Hodgkin Lymphoma

**DOI:** 10.1371/journal.pone.0077409

**Published:** 2013-10-02

**Authors:** Marc A. Kowalkowski, Martha P. Mims, E. Susan Amiran, Premal Lulla, Elizabeth Y. Chiao

**Affiliations:** 1 Department of Medicine, Baylor College of Medicine, Houston, Texas, United States of America; 2 Houston Health Services Research and Development Center of Excellence, Michael E. DeBakey Veterans Affairs Medical Center, Houston, Texas, United States of America; 3 Department of Pediatrics, Baylor College of Medicine, Houston, Texas, United States of America; 4 Dan L. Duncan Cancer Center, Baylor College of Medicine, Houston, Texas, United States of America; IRCCS National Cancer Institute, Italy

## Abstract

**Background:**

The incidence of Hodgkin lymphoma (HL) has increased since introduction of combined antiretroviral therapy (cART). While HIV-related HL is highly associated with EBV, the causes underlying the rising incidence remain unclear. The aim of this study was to evaluate the effect of immune reconstitution on HL incidence among a cohort of HIV-infected male veterans ever receiving cART.

**Methods:**

We performed a retrospective cohort study utilizing data from the Veterans Affairs HIV Clinical Case Registry from 1985-2010. HL cases were identified using ICD-9 codes (201.4-9). Poisson regression was conducted to evaluate relationships between cART-related immunologic measures (e.g., nadir CD4 before cART, time-updated CD4, % time undetectable HIV RNA) and HL incidence. Additionally, we examined CD4 change after cART initiation.

**Results:**

31,056 cART users contributed 287,256 person-years and 196 HL cases (IR=6.8/10,000 person-years). Rate of CD4 increase after cART was worse among HL cases than non-cases (p<0.05). In multivariate regression, HL risk was elevated among veterans with recent CD4 200-350 cells/µL (IRR=1.67, 95%CI=1.16-2.40) and <200 cells/µL (IRR=1.61, 95%CI=1.09-2.39), compared to >350 cells/µL. HL risk was lower among veterans with >80% time undetectable HIV RNA (IRR=0.57, 95%CI=0.35-0.92) and 40-80% undetectable (IRR=0.68, 95%CI=0.47-0.99), compared to <40% undetectable. HL risk was higher in the first 12 months (IRR=2.02, 95%CI=1.32-3.10) and 12-24 months (IRR=1.75, 95%CI=1.16-2.64) after cART initiation, compared to >36 months.

**Conclusion:**

These data highlight immunosuppression and poor viral control may increase HL risk, specifically during immune reconstitution in the interval post cART initiation. Findings suggest an immune reconstitution type mechanism in HIV-related HL development.

## Introduction

The introduction of combination anti-retroviral therapy (cART) in 1996 transformed the epidemiology of HIV infection and prolonged survival of infected individuals in the United States [1,2]. However, during this interval, the incidence of non-AIDS-defining malignancies (NADMs) has increased [3-5]. Hodgkin lymphoma (HL) is one of the most commonly-diagnosed NADMs among HIV-infected persons [6,7]. Previous studies have demonstrated that HL risk is over 10-fold higher among HIV-infected individuals than the general population [8-12], and have also shown HL incidence to be increasing in the cART era [4,13].

Although HIV-related HL has been strongly linked to the Epstein-Barr virus (EBV), the causes for the increased HL incidence in the cART era remain unclear [14]. Previous studies have described the complex influence of immune function in mediating HL development in this population; however, these studies have been limited by relatively small samples, and findings have been largely inconsistent [10,15-17]. Furthermore, there is limited research examining HIV-related immune factors and immune reconstitution associated with increased HL incidence in a cohort of individuals receiving cART. The aim of the present study was to evaluate the effect of immune reconstitution on HL incidence among a cohort of male US military veterans ever receiving cART and diagnosed with HIV infection between 1985 and 2010.

## Methods

This study involved the use of secondary data from the Department of Veterans Affairs (VA) HIV Clinical Case Registry (CCR). The risk to the individual subject was no more than minimal, and the requirement for informed consent was waived. The informed consent waiver and study protocol was approved by the Institutional Review Board of Baylor College of Medicine.

### Database

The Veterans Health Administration has the largest integrated healthcare system and is the largest provider of HIV care in the United States [18]. The VA HIV CCR is a nationwide registry containing health-related information on all known HIV-infected VA users [18,19]. Established in 1992, the registry draws upon the electronic medical records of over 60,000 HIV-infected patients cared for by the VA since the registry’s inception. The registry includes demographic, laboratory, pharmacy, outpatient clinic visit, hospitalization data, and dates of death.

### Inclusion Criteria

**Figure 1:**
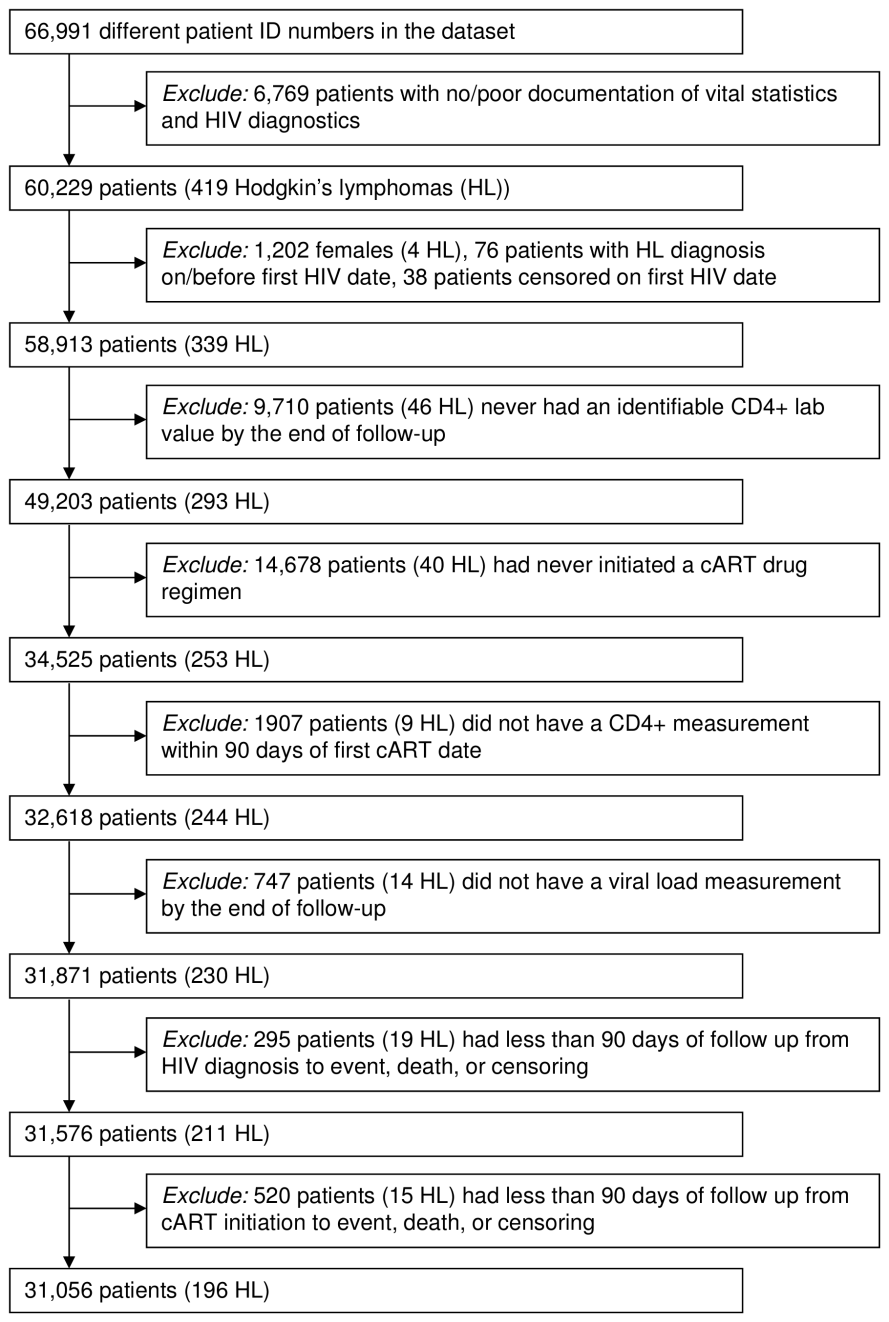
Flow chart of selection criteria to generate final cohort of HIV-infected veterans.

The study population was restricted to HIV-infected veterans over the age of 18 with at least one CD4 count and HIV RNA measurement (see Figure 1). Inclusion required an identifiable HIV diagnosis date, based on the earliest *International Classification of Diseases, Ninth Revision* (ICD-9) code for HIV, the earliest positive HIV-related test (e.g., ELISA, Western Blot, HIV RNA), or the earliest antiretroviral therapy pharmacy record. Individuals with only a single ICD-9 code for HIV and no lab or pharmacy records were removed. Due to the limited number of females (<2%), only male veterans were included in our analyses. Additionally, we removed individuals whose date of death or censor was the same as their initial HIV diagnosis date.

We included only individuals ever receiving cART, defined as any combination of 2 nucleoside reverse transcriptase inhibitors classes and 1 of either non-nucleoside reverse transcriptase inhibitor, protease inhibitor classes, integrase inhibitors, or CCR5 inhibitors and any combination of two classes. From cART users, we excluded individuals without documented CD4 count or HIV RNA measurements within 90 days of cART initiation. Finally, we excluded individuals diagnosed with HL, or who were censored or died prior to or within 90 days of cART initiation.

### Primary Outcome

The primary outcome was incident HL, based on ICD-9 codes (201.4-9). Prevalent HL cases (i.e., individuals diagnosed before or within 6 months after the initial HIV diagnosis date) were excluded. The follow-up interval for longitudinal analyses spanned from the index visit to HL diagnosis, death or December 31, 2010 (the final date of the current CCR iteration), whichever occurred first.

### Data management and definitions

To account for potential differences in the frequency of follow-up visits for registry patients, each individual’s follow-up duration was divided into 7-day intervals. Laboratory values were updated at the beginning of each interval, with the last observation carried forward when no new measurement was available. Immune function prior to cART was estimated from the nadir CD4 count over the interval from HIV diagnosis to cART initiation. For individuals who received cART on the same date as the HIV diagnosis, the initial CD4 count was captured as the nadir. Time-updated recent CD4 count was included to monitor the effect of fluctuations in immune status throughout the follow-up period. Measurement of recent CD4 count incorporated a 6-month time lag to avoid capturing immunodeficiency secondary to undetected malignancy in the interval prior to HL diagnosis.

We generated a time-dependent metric to measure the cumulative percentage of follow-up time that an individual’s HIV RNA measurement was in the undetectable range. For standardization of operational procedures at different contributing VA facilities over all study years, the value for undetectable HIV RNA was established as <500 copies/cell. The percent time undetectable HIV RNA was modeled as <40%, 40-80%, and >80% time. The time from cART initiation to HL, censor, or death was categorized as <12 months, 12-24 months, 24-36 months and >36 months.

Additional covariates included age at HIV diagnosis and race/ethnicity. To capture health severity and non-HIV comorbid conditions, we applied the Deyo modification of the Charlson comorbidity index, a measure developed to predict 10-year mortality [20,21]. We excluded points allotted for diagnosis of HIV infection. Time from HIV diagnosis to cART initiation was categorized as <5 years, 5-10 years, or >10 years.

### Statistical Analysis

All analyses were performed using SAS^®^ version 9.1 (SAS Institute, Cary, NC). We calculated the incidence of HL by dividing the number of HL cases by person-years of follow-up. Incidence rates were estimated in the overall study cohort and in each stratum defined by: age at HIV diagnosis, race/ethnicity, illicit drug use, Deyo comorbidity score, time from HIV diagnosis to cART, time-updated CD4, % time undetectable HIV RNA, and time after cART initiation. From these estimates, stratum-specific incidence rate ratios were also calculated.

**Figure 2:**
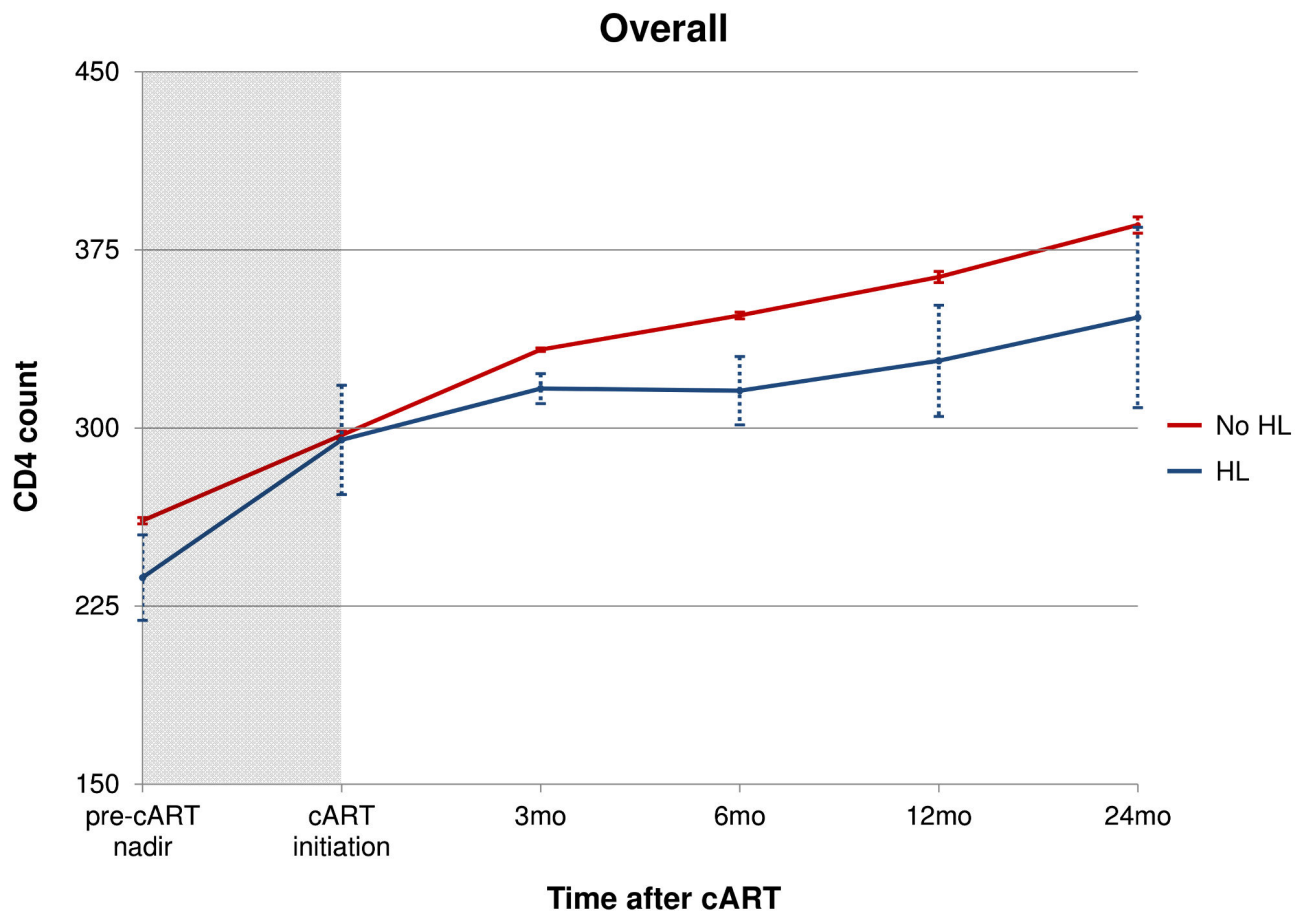
Change in CD4 count after cART initiation stratified by Hodgkin lymphoma (HL) status. *Note*: Hodgkin Lymphoma (HL) cases diagnosed after 24 months of cART initiation were included to avoid capturing CD4 change secondary to undiagnosed HL. Dotted lines show standard error.

**Figure 3:**
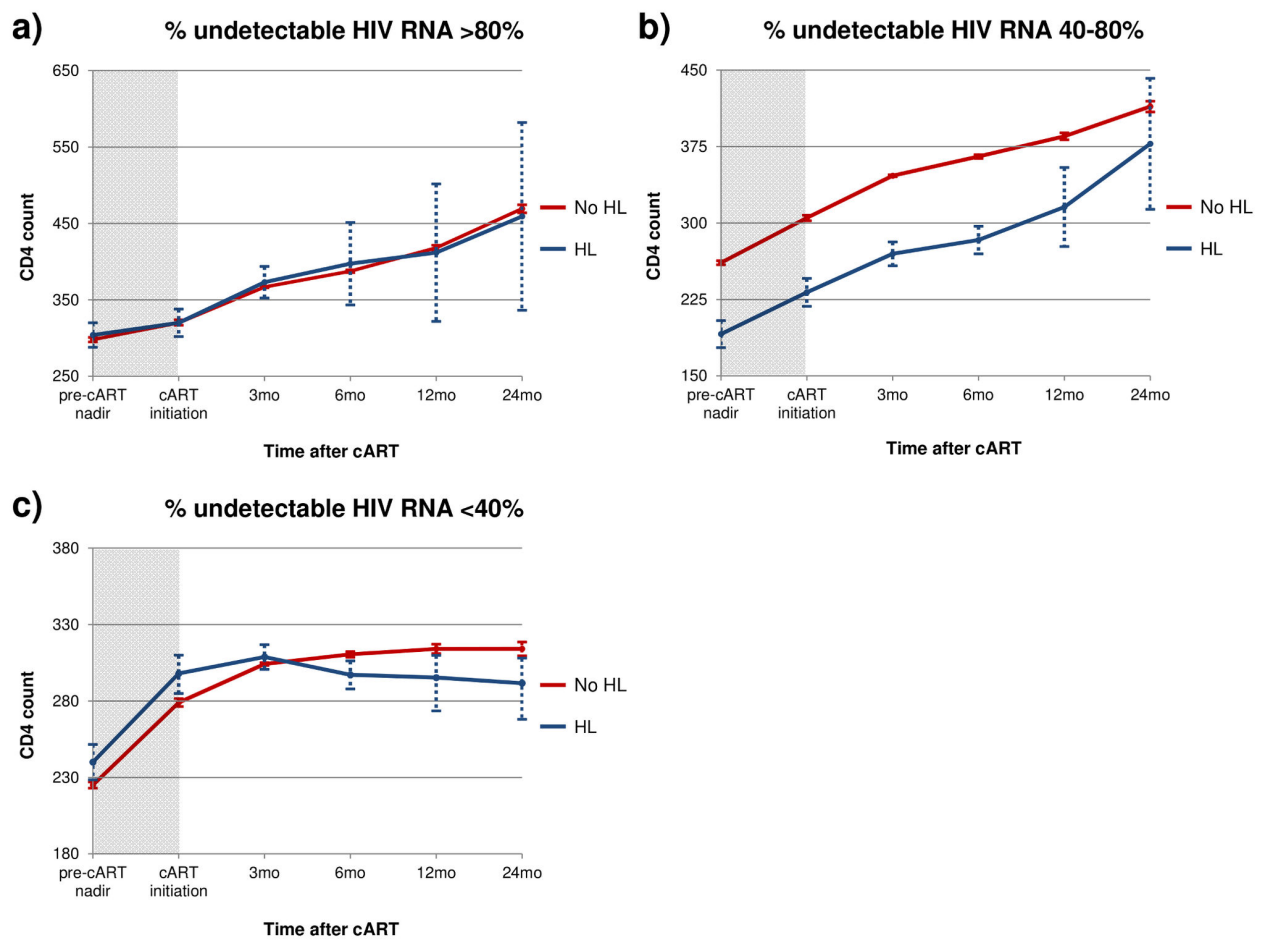
Change in CD4 count after cART initiation in groups of HIV-infected individuals with different percent time undetectable HIV RNA (a) Individuals with >80% time undetectable HIV RNA (b) Individuals with 40-80% time undetectable HIV RNA (c) Individuals with <40% time undetectable HIV RNA. *Note*: Hodgkin Lymphoma (HL) cases diagnosed after 24 months of cART initiation were included to avoid capturing CD4 change secondary to undiagnosed HL. Dotted lines show standard error.

Change in CD4 count was calculated from cART initiation to 3-, 6-, 12-, and 24-months after beginning therapy. The rate of CD4 change was compared between HL cases and non-cases. Specific to the change in CD4 count analyses (Figures 2 and 3), we excluded incident HL cases diagnosed within the first 24 months after beginning cART in order to mitigate potential inclusion of CD4 count decreases secondary to latent or recently diagnosed HL. Additional subgroup analyses were conducted among individuals with percent time undetectable HIV RNA load <40%, 40-80%, and >80% over the follow-up interval.

Poisson regression models were constructed to evaluate the impact of cART-related factors (e.g., CD4 counts, percent time undetectable HIV RNA, and time after cART initiation) on HL incidence among cART users. To control for potential confounding, multivariate Poisson regression models were fit and adjusted for clinically-relevant covariates described previously (i.e., age at HIV diagnosis, race/ethnicity, illicit drug use, Deyo comorbidity score, and time from HIV diagnosis to cART initiation). Several sensitivity analysis were conducted, including a multivariate model wherein nadir and recent CD4 measures were replaced with the measure of an individual’s % time with CD4 <350 cells/µL. Additional sensitivity analyses were conducted to verify study findings in separate, restricted cohorts excluding individuals who 1) died and were no longer at risk to develop HL or 2) were diagnosed with HIV before cART introduction in 1996 to account for the different treatments available to infected individuals during these time intervals.

## Results

In our sample, 31,056 HIV-infected male veterans contributed 287,256 person-years from 1985-2010. Frequency distributions for cohort characteristics are shown in Table 1 along with the crude incidence overall and in subgroups. The median age at HIV diagnosis was 44 years (range=18-89 years). Non-Hispanic African Americans were the largest racial/ethnic group (49%). Approximately 35% of individuals had documented use of illicit drugs. Additionally, 82% of the cohort received cART within 5 years of HIV diagnosis.

**Table 1:** Characteristics of 31,056 individuals ever receiving cART.

	**N (%)**	**PY**	**HL**	**IR**
**Overall**	31056	287256	196	6.82
**Age** (years)				
< 30	2089 (6.7)	23429	12	5.12
30-39	8054 (25.9)	92696	60	6.47
40-49	11786 (38.0)	110329	70	6.34
50-59	6518 (21.0)	45805	40	8.73
60 and above	2609 (8.4)	14996	14	9.33
**Race/ethnicity**				
White	11881 (38.3)	111903	76	6.79
African American	15333 (49.4)	142981	95	6.64
Hispanic	2363 (7.6)	23285	23	9.88
Unknown/Other	1479 (4.8)	9087	2	2.20
**Time from HIV diagnosis to cART** (years)				
< 5	25537 (82.2)	204642	162	7.92
5-10	4359 (14.0)	62680	28	4.47
> 10	1160 (3.8)	19933	6	3.01
**Illicit drug use**				
No	20301 (65.4)	172848	136	7.87
Yes	10755 (34.6)	114407	60	5.24
**DEYO co-morbidity score**				
2 and above	5718 (18.4)	58937	20	3.39
1	9157 (29.5)	96438	44	4.56
0	16181 (52.1)	131881	132	10.01
**Nadir CD4 count prior to cART initiation**				
CD4 > 350	8412 (27.1)	77209	44	5.70
CD4 200-350	8588 (27.6)	83713	54	6.45
CD4 < 200	14056 (45.3)	126334	98	7.76
**CD4 count at event/censor**				
CD4 > 350	17291 (55.7)	169451	77	4.54
CD4 200-350	5790 (18.6)	53607	58	10.82
CD4 < 200	7971 (25.7)	64192	57	8.88
**% time undetectable HIV RNA at event/censor**				
< 40%	12959 (41.7)	125656	128	10.19
40-80%	10068 (32.4)	104827	44	4.20
> 80%	8029 (25.9)	56773	24	4.23
**Time after cART to event/censor** (months)				
> 36	24290 (78.2)	264104	110	4.17
24-36	2201 (7.1)	9525	22	23.10
12-24	2478 (8.0)	8354	32	38.31
<12	2087 (6.7)	5273	32	60.69

PY=Person-years; HL=Hodgkin lymphoma; IR=Incidence rate (per 10,000 PY)

The median number of CD4 and HIV RNA measurements per year was 3, respectively (both intraquartile ranges=2-4; not shown). Prior to initiating cART, 45% had a nadir CD4 <200 cells/µL. At the time of the last recorded follow-up measurement (either date of HL diagnosis, death, or censoring), 56% had a CD4 count above 350 cells/µL, while 26% were below 200 cells/µL. Additionally, 42% of individuals had <40% time undetectable HIV RNA over their follow-up interval.

During the study interval, 196 HL cases were identified. HL incidence among the entire cohort was 6.8/10,000 person-years (PY) (95% CI=5.8-7.8/10,000 PY). In subgroup analyses, HL incidence was highest among those <12 months after initiating cART (IR=60.7/10,000PY).

Table 2 shows the results of the multivariate Poisson regression models conducted to evaluate the effects of each of four cART- and immune-related factors, adjusted for age at HIV diagnosis, race/ethnicity, illicit drug use, time from HIV diagnosis to cART initiation, and Deyo comorbidity score. Risk for developing HL was incrementally higher during the time intervals closer to cART initiation (*p*-trend<0.001). Compared to time greater than 3 years, HL incidence was significantly higher in the first year (IRR=2.02, 95%CI=1.32-3.10) and in the second year (IRR=1.75, 95%CI=1.16-2.64) following cART initiation. HL risk also remained elevated in veterans with a recent CD4 <200 cells/µL (IRR=1.61 (1.09-2.39) and 200-350 cells/µL (IRR=1.67, 95%CI=1.16-2.40), compared to CD4 >350 cells/µL. Veterans with a greater percentage of follow-up time with an undetectable HIV RNA load had reduced HL risk. HL risk was lowest among veterans with >80% time undetectable HIV RNA, relative to veterans with <40% time undetectable HIV RNA (IRR=0.57, 95%CI=0.35-0.92). Results from the multivariate models were confirmed in separate sensitivity analyses restricted to veterans diagnosed with HIV in the cART era (1996-2010) and veterans who did not die over the study interval (not shown). Results from the sensitivity analysis assessing HL risk in a multivariate model wherein nadir and recent CD4 were replaced with % time CD4 <350 cells/µL are shown in Appendix S1. Results do not differ from the findings shown for the multivariate model in Table 2 but do indicate that individuals with >80% time CD4 <350 had increased HL risk (IRR=1.57, 95%CI=1.13-2.17), compared to individuals with <40% time CD4 <350.

**Table 2:** Multivariate analysis of Hodgkin’s lymphoma incidence among HIV-infected male veterans ever receiving cART.

	**Unadjusted IRR (95%CI)**	**Adjusted IRR (95%CI)**
**Age at HIV diagnosis**		
Age (continuous)	1.01 (0.99-1.02)	1.01 (0.99-1.02)
**Race/ethnicity**		
White	1	1
African American	0.98 (0.72-1.32)	0.99 (0.72-1.36)
Hispanic	1.45 (0.81-2.31)	1.48 (0.92-2.40)
Other	0.32 (0.09-1.23)	0.32 (0.08-1.31)
**Illicit drug use**		
No	1	1
Yes	0.67 (0.49-0.90)	0.94 (0.62-1.27)
**Time from HIV diagnosis to cART** (years)		
<5	1	1
5-10	0.56 (0.38-0.84)	0.81 (0.36-1.86)
>10	0.38 (0.17-0.83)	0.82 (0.53-1.25)
**DEYO co-morbidity score**		
2 and above	1	1
1	1.34 (0.79-2.28)	0.89 (0.52-1.53)
0	2.95 (1.88-4.62)	0.98 (0.60-1.61)
**Nadir CD4 count prior to cART**		
CD4 > 350	1	1
CD4 200-350	1.13 (0.76-1.68)	0.99 (0.67-1.46)
CD4 < 200	1.36 (0.95-1.94)	1.05 (0.70-1.57)
**Recent CD4 count**		
CD4 > 350	1	1
CD4 200-350	2.38 (1.71-3.31)	1.67 (1.16-2.40)
CD4 < 200	1.95 (1.40-2.73)	1.61 (1.09-2.39)
**% time undetectable HIV RNA**		
< 40%	1	1
40-80%	0.41 (0.30-0.57)	0.68 (0.47-0.99)
> 80%	0.42 (0.27-0.63)	0.57 (0.35-0.92)
**Time after cART initiation** (months)		
>36	1	1
24-36	5.55 (3.69-8.32)	1.27 (0.79-2.04)
12-24	9.20 (6.66-12.71)	1.75 (1.16-2.64)
<12	14.57 (10.83-19.60)	2.02 (1.32-3.10)

IRR = Incidence rate ratio

Retrospective analysis of CD4 restoration over the first two years following cART initiation showed different rates of change between HL cases and non-cases (Figure 2). During the first 3 months of cART, both HL cases and non-cases experienced similar rates of CD4 restoration, approximately 30-40 cells/µL. Although, non-cases continued to exhibit increasing rates of CD4 counts over the remainder of the two-year interval, a small decline in CD4 counts was observed among HL cases. Subgroup analysis is shown in Figure 3. Figures 3a and 3b show, in groups of HIV-infected individuals with >80% and 40-80% time undetectable HIV RNA, HL cases and non-cases experienced steady and continual increasing rates of CD4 expansion following cART initiation. In Figure 3b, among those with moderate HIV control (i.e., % time undetectable HIV RNA 40-80%), HL cases had significantly lower CD4 counts at cART initiation. However, the rate of CD4 increase was similar between HL cases and non-cases. Figure 3c illustrates that individuals with <40% time undetectable HIV RNA had the poorest levels of CD4 restoration and, after an initial period of CD4 repopulation, HL cases experienced declining CD4 counts. From 3-24 months after cART initiation, different CD4 trajectories were observed between HL cases and non-cases; specifically, non-cases continued to show slight CD4 increases while HL cases experienced declining CD4 to near cART initiation levels.

## Discussion

To our knowledge, this is the first study to conduct a careful analysis of cART-associated immune reconstitution on the risk of HL, exclusively among a cohort of HIV-infected veterans who had all received cART. We were able to determine that HL risk was reduced among individuals with a greater percent time undetectable HIV RNA, and that HL risk was elevated in the interval directly following cART initiation, after adjusting for nadir and recent CD4 counts. Furthermore, we found significant differences in the rate of CD4 repopulation after cART initiation between HL cases and non-cases, specifically among HIV-infected individuals with poor control of HIV RNA load.

Previous studies have primarily assessed HL in association with individual or time-updated measurements of HIV RNA, and did not find a direct relationship. For example, Bohlius et al [10] did not find a statistically significant effect of time-updated HIV RNA on HL risk. In the current sample of more than 30,000 HIV-infected veterans receiving cART, our findings suggest valuable information may be obtained by incorporating cumulative metrics of HIV exposure, calculated via serial measurement, compared to a single viral load measurement.

In addition, other studies have observed a range of associations between different categories of CD4 counts and HL risk, including elevated risk at levels of both severe and moderate immune suppression [15-17]. In a previous time-updated analysis of recent CD4 counts, Bohlius et al [10] reported that HL risk was associated with CD4 depletion in the year prior to HL diagnosis. Our findings suggest HL risk was also elevated among individuals with recent CD4 counts below 350 cells/µL but was highest in moderately immune suppressed individuals (i.e., CD4 200-350 cells/µL) [14]. Malignant HRS cell proliferation is reliant upon interaction with an assortment of benign immune cells [22]. Our results support the hypothesis that moderate immune competence after cART initiation may provide a more adequate immune response and suitable environment to support HL development [23,24].

We also found that HL risk was more than two-fold higher in the first year following cART initiation, compared to more than 3 years of follow-up. There has only been one previous study examining HL risk following cART initiation [25], which demonstrated that HL risk was nearly 3-fold higher in the first 3 months after cART initiation. However, these results were not statistically significant in a multivariate analyses adjusted for additional epidemiologic factors, including nadir and recent CD4 counts. We hypothesize that we were able to demonstrate a statistically significant difference in our multivariable model due to the large number of HL, which allowed for increased power in the current analysis. Indeed, our current findings corroborate the results reported by Lanoy et al [25] but are robust to multivariate adjustment.

We observed significant differences retrospectively in the rate of change in CD4 count in the first two years after cART initiation between HL cases and non-cases. Our results indicated a divergent trend in the increase in CD4 counts, beginning between 3-6 months after cART initiation. Over the first 3 months following cART initiation, HL cases and non-cases experienced similar rates of CD4 repopulation. However, from 3-6 months after cART initiation, HL cases, on average, experienced a brief interval of declining CD4 counts, while non-cases experienced continual CD4 proliferation. When stratified by % time undetectable HIV RNA, a declining rate of CD4 change was only observed among HL cases in the subgroup with poor control of HIV RNA load (i.e., <40% time undetectable HIV RNA). Our results from this subgroup analysis support previous findings of declining CD4 counts among HL cases following cART initiation, but demonstrate that those individuals with poor HIV viral load control appear to have the greatest rate of CD4 count decline [10].

Because HIV-related HL are highly associated with EBV [26,27], it is of note that our results may support evidence from previous studies correlating the effect of HIV viral control on EBV replication. A previous prospective study of 20 HIV-infected individuals followed for 5 years reported individuals with an optimal HIV response following cART initiation (i.e., nearly all HIV viral loads were undetectable) experienced significant CD4 restoration. In this group of individuals, EBV DNA load levels were restored to near normal, healthy control levels within 1-2 years [28]. However, individuals with persistent detectable HIV viral load levels after cART initiation did not experience concurrent decreasing EBV DNA loads. Similarly, Righetti et al [29] demonstrated that individuals with an incomplete virological response to cART experienced a significant increase in EBV latent membrane protein 1. Thus, individuals with persistent detectable HIV viral load after cART may have an elevated EBV load and subsequent elevated HL risk over time. It is possible that lymphomagenesis may be associated with rising levels of cell-associated EBV, which in turn has been shown to be coupled with concurrent increased EBV DNA in plasma and immunoglobulin levels, indicating B-cell stimulation [30].

It is likely that multiple factors act in combination during the early treatment interval. Results from the current study support a hypothesis that increased HL risk occurs during an interval that EBV loads may remain elevated following cART initiation, in spite of immune reconstitution. EBV levels may remain high until adequate long-term control of HIV viral load is achieved. Friis et al [28] suggested that it may take 1-2 years or longer following cART initiation to achieve effective EBV control, exactly mirroring the period of time that HL risk was highest in the current sample. Additionally, our findings may support the intriguing hypothesis that this increased HL risk in the early treatment interval may be related to an EBV mediated immune reconstitution mechanism.

It is possible that although CD4 repopulation following cART may provide added protection against HIV replication, this repopulation also may present additional targets for viral infection [31]. Mahnke et al [32] reported that developing immune reconstitution inflammatory syndrome (IRIS) following cART was characterized by expansion of highly-activated, highly-differentiated, polyfunctional CD4 cells specific to the underlying IRIS-associated antigen, accompanied by increased levels of proinflammatory cytokines. Immune dysregulation during IRIS has been hypothesized to originate from a combination of poor IRIS-associated antigen clearance secondary to HIV infection [33] and CD4 repopulation following cART [34]. In the context of EBV infection and HL development, EBV-specific CD4 cell responses rapidly decline with HIV co-infection, dampening EBV clearance. Subsequent repopulation of EBV-specific CD4 cells under poorly regulated, proinflammatory immune conditions may yield an environment conducive to HRS cell activation and HL development. Additional research is needed to confirm the detailed mechanisms impacting HL development after cART.

The findings from the current study should be viewed within the context of the study design. Data for the present retrospective cohort study was extracted from a national, system-wide VA registry of HIV-infected veterans. Certain limitations are inherent in large registry databases, such as potential variations in the frequency of follow-up visits within the data collection period. However, we observed similar frequencies of CD4 count and HIV RNA measurements per person-year of follow-up, and we attempted to reduce the potential for information bias by segmenting follow-up into one-week intervals, thus establishing equivalent visit frequencies per individual. In addition, ICD-9 diagnostic codes for HL have not been previously validated in this database. However, diagnostic codes for other malignancies have been successfully validated within the VA [35,36]. Finally, this study was conducted exclusively on a population of considerable public health interest, male military veterans, which may have implications on the generalizability of findings to other populations.

Our findings support previous indications that an immune reconstitution mechanism may play a key role in HIV-related HL development. Additionally, findings indicate immune and virologic markers may provide significant predictors of HL risk and may be useful in initiating HL screening. Individuals with poor control of HIV replication while on cART represent a high-risk subgroup. Future research evaluating the role of EBV infection in the etiology of HIV-related HL and the interaction between EBV and active HIV viral replication are needed.

## Supporting Information

Appendix S1 **Multivariate analysis of Hodgkin’s lymphoma incidence among HIV-infected male veterans ever receiving cART, including % time CD4 <350 cells/µL.**
(DOC)
