# Correction: Effect of Immune Reconstitution on the Incidence of HIV-Related Hodgkin Lymphoma

**DOI:** 10.1371/annotation/750f93e7-3fee-4ae0-81e4-058787a9e9a1

**Published:** 2013-10-15

**Authors:** Marc A. Kowalkowski, Martha P. Mims, E. Susan Amirian, Premal Lulla, Elizabeth Y. Chiao

The name of the third author was spelled incorrectly. 

The correct name is: E. Susan Amirian. 

The correct citation is: Kowalkowski MA, Mims MP, Amirian ES, Lulla P, Chiao EY (2013) Effect of Immune Reconstitution on the Incidence of HIV-Related Hodgkin Lymphoma. PLoS ONE 8(10): e77409. doi:10.1371/journal.pone.0077409. 

