# Supplementary material for: Effect of Immune Reconstitution on the Incidence of HIV-Related Hodgkin Lymphoma
Source: PLoS One. 2013 Oct 2;8(10):e77409. doi: 10.1371/journal.pone.0077409 (PMC3788758; doi:10.1371/journal.pone.0077409)
Supplement: Appendix S1 — Multivariate analysis of Hodgkin’s lymphoma incidence among HIV-infected male veterans ever receiving cART, including % time CD4 <350 cells/µL. (DOC) [file pone.0077409.s001.doc]

**Appendix S1:** Multivariate analysis of Hodgkin’s lymphoma incidence among HIV-infected male veterans ever receiving cART, including % time CD4 <350 cells/μL

|  | **Adjusted IRR (95%CI)** |
| --- | --- |
| **Age at HIV diagnosis** |  |
| Age (continuous) | 1.01 (0.99-1.02) |
| **Race/ethnicity** |  |
| White | 1 |
| African American | 0.98 (0.71-1.35) |
| Hispanic | 1.48 (0.91-2.40) |
| Other | 0.32 (0.08-1.31) |
| **Illicit drug use** |  |
| No | 1 |
| Yes | 0.95 (0.63-1.28) |
| **Time from HIV diagnosis to cART** (years) |  |
| <5 | 1 |
| 5-10 | 0.82 (0.36-1.86) |
| >10 | 0.82 (0.53-1.25) |
| **DEYO co-morbidity score** |  |
| 2 and above | 1 |
| 1 | 0.90 (0.52-1.54) |
| 0 | 0.99 (0.60-1.62) |
| **% time CD4 count <350 cells/μL** |  |
| < 40% | 1 |
| 40-80% | 1.37 (0.91-2.04) |
| > 80% | 1.57 (1.13-2.17) |
| **% time undetectable HIV RNA** |  |
| < 40% | 1 |
| 40-80% | 0.66 (0.46-0.96) |
| > 80% | 0.55 (0.34-0.87) |
| **Time after cART initiation** (months) |  |
| >36 | 1 |
| 24-36 | 1.26 (0.79-2.03) |
| 12-24 | 1.74 (1.16-2.63) |
| <12 | 2.01 (1.32-3.08) |

IRR=Incidence rate ratio
